# Extracranial metastatic oligodendroglioma with molecular progression, case presentation

**DOI:** 10.1186/s13000-024-01529-7

**Published:** 2024-07-26

**Authors:** Nour Kurdi, Attila Mokánszki, Ingrid Balogh, Anikó Ujfalusi, Sándor Szabó, Gábor Méhes, Judit Bedekovics

**Affiliations:** 1 Department of Pathology, Faculty of Medicine, University of Debrecen, Nagyerdei krt 98, Debrecen, H-4032 Hungary; 2 Department of Oncology, Faculty of Medicine, University of Debrecen, Debrecen, Hungary; 3 Department of Laboratory Medicine, Faculty of Medicine, University of Debrecen, Debrecen, Hungary; 4 Department of Neurosurgery, Faculty of Medicine, University of Debrecen, Debrecen, Hungary

**Keywords:** Oligodendroglioma, Extraaxial metastasis, IDH mutation, Bone marrow, Molecular progression

## Abstract

**Background:**

Extraneural metastasis of central nervous system tumors is generally rare and most often reported in glioblastomas and medulloblastomas, whereas oligodendrogliomas seem to have the lowest risk of extracranial metastasis. Given its infrequent occurrence, both the diagnosis and therapy of metastatic oligodendroglioma is often challenging.

**Case presentation:**

This case study presents an oligodendroglioma, the isocitrate dehydrogenase 1 (IDH1) mutant, 1p/19q-codeleted tumor with bone marrow metastasis. The significance of this case lies in the comprehensive molecular analysis conducted for both the primary tumor and the metastasis. Chromosome 7 trisomy and chromosome 10 monosomy (+ 7/-10) were detected in the metastasis indicating molecular progression, which, to the best of our knowledge, has not been previously documented in metastatic oligodendroglioma.

**Conclusions:**

This case study serves additional information for better understanding of the metastatic capabilities of CNS tumors.

## Background

Oligodendroglioma is an adult-type, diffuse glioma, comprising about 5% of primary intracranial tumors. Oligodendroglioma is molecularly defined by the presence of the isocitrate dehydrogenase 1 (IDH1) (most commonly IDH1 p.R132H*)* or *IDH2* mutations and 1p/19q codeletion, frequently with an additional Telomerase Reverse Transcriptase (TERT) promoter mutation. Most IDH mutant and 1p/19q-codeleted oligodendrogliomas show a clear predilection for frontal lobe localization, often in the cortex along the gray matter–white matter boundary. Histologically, classic oligodendroglioma cells are characterized by a “fried-egg” microscopic appearance composed of spherical nuclei surrounded by clear cytoplasm and perinuclear halo as well as a “chicken-wire” blood vessel pattern within the tumor is also characteristic. The 5th edition of classification of central nervous system (CNS) tumors by the World Health Organization (WHO) distinguishes two oligodendroglioma subtypes based on the grade: oligodendroglioma, CNS WHO grade 2, and oligodendroglioma, CNS WHO grade 3. Similarly to other diffusely infiltrative gliomas, oligodendrogliomas may extend into adjacent brain matter in a gliomatosis cerebri pattern and in late-stage disease and distant leptomeningeal involvement may occur [1, 2, 3].

While extraneural metastasis of a primary CNS neoplasm is rare in general, oligodendrogliomas seem to have the lowest risk of extracranial metastasis as seen in a 1979 reported series of 116 cases of CNS tumors with extracranial metastases, of which oligodendrogliomas represented 5.25% [4]. In an extensive literature review of metastatic oligodendrogliomas, bone and bone marrow was the most frequent extracranial metastatic area [5]. While the aforementioned literature reviews have revealed critical insight into such a rare phenomenon, significant advancements have occurred in the methodology of diagnostic and prognostic assessment of metastatic oligodendrogliomas since that time. Despite numerous reported cases of metastatic oligodendrogliomas, most lack molecular confirmation of both primary and metastatic lesions. This is especially significant for cases diagnosed using pre-2016 WHO CNS4 criteria, which may lead to the inclusion of oligodendroglioma-like glioblastomas. This case report aims to provide detailed molecular characterization of an oligodendroglioma with bone marrow metastasis.

## Case presentation

### Clinical summary

**Fig. 1:**
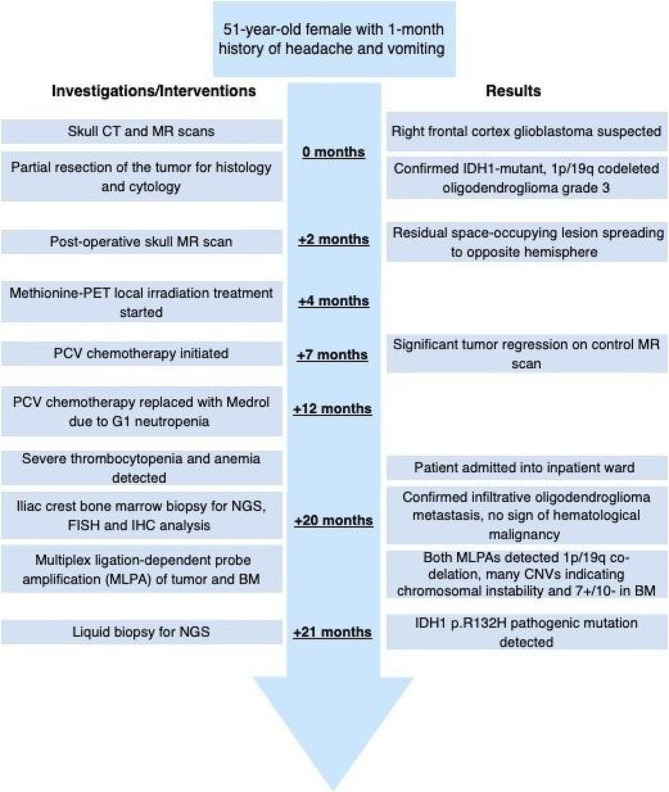
Timeline of the disease history, diagnostic evaluations and management. PCV: procarbazine, lomustine (CCNU) and vincristine; BM: bone marrow; NGS: next generation sequencing; CNV: copy number variation

A 51-year-old woman presented with complaints of a headache and vomiting for the past month. Skull CT and MR scans revealed a mass in the right frontal cortex. Control chest, abdomen, and pelvic CT examinations were performed to rule out another primary tumor, and no other space occupying lesion was detected. Partial resection of the right frontal tumor was performed. The integrated diagnosis was oligodendroglioma IDH mutant, 1p/19q codeleted, CNS WHO grade 3. No further molecular analysis was performed at the time of diagnosis. Postoperative skull MR revealed development of right frontal and temporal arachnoid cysts/ tumor with perilesional cyst formation. The patient received focal brain irradiation therapy based on methionine PET planning which was followed by procarbazine, lomustine, vincristine (PCV) chemotherapy. The patient was treated with methionine-PET based local irradiation which was followed by PCV chemotherapy. Control skull MR showed residual tumor with spread to the opposite hemisphere and perifocal edema. Five months later PCV treatment was discontinued due to persistent grade 1 neutropenia and the patient was started on Medrol instead. Neurosurgery was not recommended at this time, however additional stereotactic radiation therapy (SRT) and Temozolomide (TMZ) treatment was recommended. Administration of TMZ chemotherapy was planned, however severe thrombocytopenia (thrombocyte count: 11 giga/L) and anemia (hemoglobin: 62 g/L) was detected in blood count and the patient was admitted into the inpatient clinic, Fig. 1. Iliac crest bone marrow (BM) biopsy was performed to test for suspected hematological malignancy. The histological evaluation revealed bone marrow metastasis of oligodendroglioma,.

## Methods

### Immunohistochemistry

Formalin fixed, paraffin embedded (FFPE) tissue blocks and four um thick tissue sections were used for immunohistochemical analysis in the Department of Pathology, University of Debrecen, Hungary. The most relevant immunohistochemical stains were the following: anti-GFAP (mouse monoclonal, clone 6F2; DAKO, Dako, Glostrup, Denmark; 1:500) anti-Olig2 (polyclonal, Cat. AB9610; Merck Life Science, Darmstadt, Germany; 1:2000), anti-IDH1 (R132H) (mouse monoclonal, clone H09; BIOZOL, Germany; 1:200), anti-S100 (polyclonal, Cat. NCL-L-S100p; Leica Biosystems, IL, USA; 1:1000), histone H3 p.K27me3 (rabbit monoclonal, clone EPR18607; Abcam Limited, Cambridge, UK; 1:50). All immunohistochemical reactions were performed according to the manufacturer instructions.

### Next-generation sequencing

Next-generation sequencing (NGS) was performed from bone marrow core biopsy and peripheral blood in the Department of Pathology, University of Debrecen, Hungary. As hematological malignancy was the expected diagnosis, bone marrow aspirate was obtained, and myeloid NGS examination was performed separately in the Laboratory Medicine Department, University of Debrecen, Hungary. For NGS library preparation from bone marrow aspirate, a DNA-based Twist Bioscience custom gene panel (Twist Bioscience, South San Francisco, CA, USA) specific for myeloid malignancies, was applied to identify the SNVs and indels. For NGS library preparation from tumor tissue-derived RNA and peripheral blood cell-free RNA samples, an RNA-based Archer FusionPlex custom gene panel (Archer DX, Boulder, CO, USA), including the IDH1 and IDH2 genes, was applied to identify the SNVs, indels, and gene fusions. This gene panel analysis 54 genes. The final libraries were quantified with a KAPA library quantification kit (Roche, Basel, Switzerland), diluted to a final concentration of 4 nM, and pooled by equal molarity. For sequencing on the MiSeq System (MiSeq Reagent kit, version 3, 600 cycles), the libraries were denatured using 0.2 nM NaOH and diluted to 40 pM with hybridization buffer (Illumina, San Diego, CA, USA). The final loading concentration was 8 pM libraries and 5% PhiX. Captured libraries were sequenced in a multiplexed fashion with a paired-end run, to obtain 2 × 150 bp reads, with a depth of coverage of at least 500×. Trimmed FASTQ files were generated using the MiSeq reporter (Illumina, San Diego, CA, USA) and were uploaded to the Archer Analysis v7 website (Archer DX, Boulder, CO, USA). For alignment, the human reference genome GRCh38 (equivalent UCSC version hg38) was built. 5% variant allele frequency was used as the cut-off value.

### Fluorescent in situ hybridization

Fluorescent in situ hybridization (FISH) was performed on 5 μm-thick sections of the FFPE block, with 1p36/1q25 and 19p13/19q13 deletion probes (MetaSystems, Altlussheim, Germany) separately, using a manufacturer’s protocol. Deparaffinized sections (Q Path Safesolv, VWR, Debrecen, Hungary) were treated with protease solution (MetaSystems, Altlussheim, Germany). Slide and probe co-denaturation was carried out at 75 °C for 10 min, while hybridization was at 37 °C in a moist chamber for 18 h (StatSpin ThermoBrite, Abbott Molecular, Des Plaines, IL, USA). After washing, the nuclei were counterstained with 4′-6′ diamidino-2-phenylindole (DAPI, MetaSystems, Altlussheim, Germany). The images were analyzed using ISIS software v.5.5.4. (MetaSystems, Altlussheim, Germany).

### Multiplex ligation-dependent probe amplification

Multiplex ligation-dependent probe amplification (MLPA) was performed using SALSA MLPA Probemix P181 Centromere mix 1 for the detection of deletions or duplications in genes close to the centromeres of all chromosomes, with the exception of the Y-chromosome (MRC-Holland, Amsterdam, The Netherlands). Amplified products were separated by size on SeqStudio genetic analyzer (Applied Biosystems, Foster City, CA, USA) and data were analyzed by the Coffalyser.Net Software (MRC-Holland, Amsterdam The Netherlands). A peak was considered abnormal when the ratio was < 0.65 (deletion) or > 1.30 (duplication) compared to the peaks of the reference probes. Genomic DNA was isolated from bone marrow using QIAamp DNA Blood Mini kit (Qiagen, Hilden, Germany). NanoDrop 2000/2000c UV–vis spectrophotometer (ThermoFisher Scientific, Waltham, Massachusetts, USA) was used to determine the quantity and quality of DNA.

## Histopathological findings

**Fig. 2:**
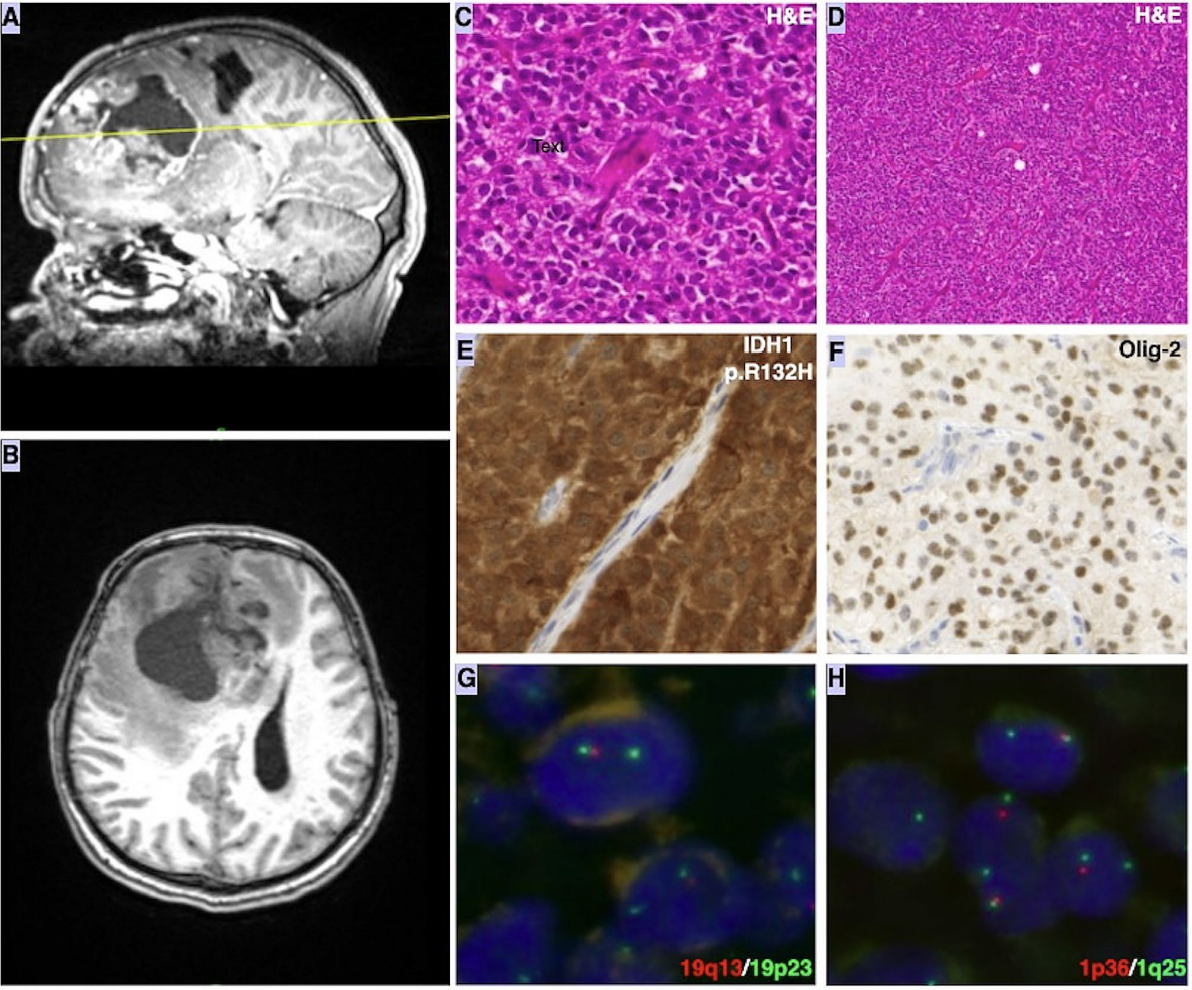
Radiologic, histologic, immunohistochemical and FISH studies of the primary oligodendroglioma from the initial brain biopsy. **A**,** B**: MR Image, 60 × 50 × 60 mm large right frontal space occupying lesion with perifocal edema; **C**,**D**: H&E staining (40X, 10X) shows a hypercellular tumor composed of sheets of cells with rounded nuclei and prominent perinuclear halos with the classic “chicken wire-like” vessels; **E**: Positive immunohistochemical staining of mutated form (R132H) of IDH1 (40X); **F**: Positive Olig-2 immunohistochemical staining; **G**,** H**: FISH with green signal indicating 19p23 and 1q25 and red signal indicate 19q13 and 1p25, revealing 1p/19q codeletion of the primary tumor sample

In the right frontal partial resection specimen microscopically, a highly cell-rich tumor was seen, composed of cells with round nuclei, lumpy chromatin and visible nucleoli with a characteristic perinuclear halo and well-circumscribed cell borders. Classic “chicken wire-like” vessels were dispersed throughout the tumor cells. Further immuno- histochemical analysis revealed diffuse GFAP+, Olig2+, IDH1 (R132H)+, hiszton H3 K27me3- immunophenotype. Additionally, retained nuclear expression of ATRX and wild type p53 expression was detected. The mitosis index was 61/10 high power field, which together with the high cellularity, indicated a higher grade. Finally, the FISH analysis revealed the 1p/19q codeletion. These findings confirmed the final diagnosis of oligodendroglioma, IDH mutant, 1p/19q codeleted, CNS WHO grade 3, Fig. 2.

**Fig. 3:**
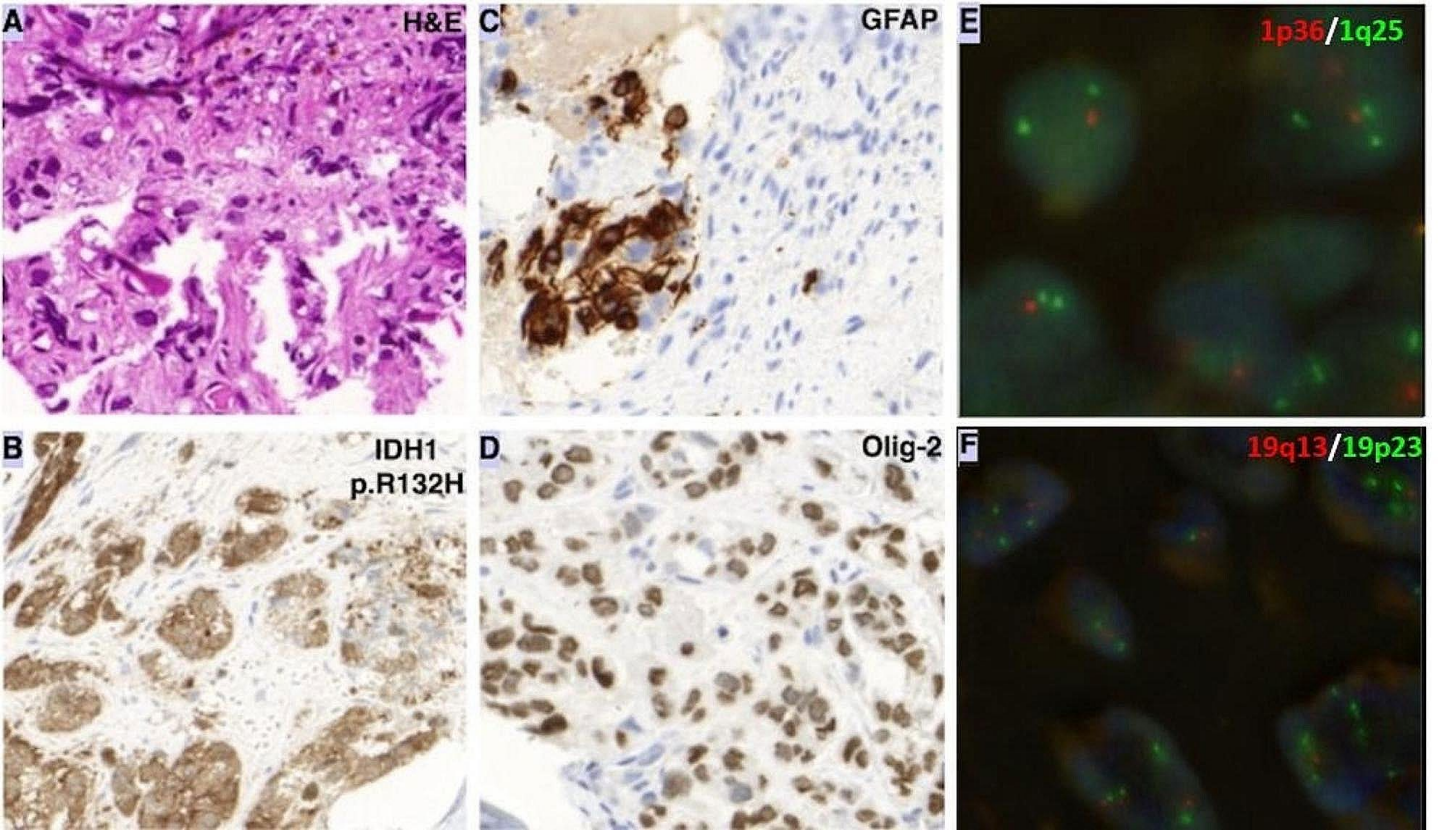
Histologic, immunohistochemical and FISH studies of the metastatic secondary oligodendroglioma from the bone marrow sample. **A**: H&E staining of the fibrotic BM samples showing small nests of atypical neoplastic cells with round nuclei and abundant cytoplasm; **B**: Immunoreactive stain for mutant IDH1 R132H; **C**: positive GFAP immunohistochemical staining; **D**: positive Olig-2 immunohistochemical staining; **E**, **F**: FISH with green signal indicating 19p23 and 1q25 and red signal indicate 19q13 and 1p25, revealing 1p/19q codeletion of the metastatic tumor cells in the BM specimen.MOLECULAR FINDINGS

In the BM core biopsy specimen massive fibrosis and near complete depletion of hematopoiesis were present. Small nests of atypical neoplastic cells with round nuclei and abundant cytoplasm were identified within the fibrotic core. Immunohistochemical analysis revealed the following immunophenotype of the neoplastic cells: GFAP+, Olig2+, IDH1 (R132H)+, S-100 protein+, MPO-, CD61-, CD71-, CK OSCAR-, CD34-, CD117-. The immunophenotype confirmed the glial origin of the atypical cells, Fig. 3. The simultaneously performed flow cytometric analyses were negative for hematological malignancies.

The diagnosis of oligodendroglioma requires the simultaneous presence of an IDH mutation and 1p/19q codeletion. The detection of these alterations was achieved using the routine gold standard methods in both the primary tumor and the BM metastasis. The IDH1 p.R132H mutation was detected by immunohistochemical examination, while the 1p/19q codeletion was confirmed with FISH analysis.

Due to the rare diagnosis, NGS analysis was performed from BM core biopsy to confirm IDH1 mutation and to detect possible further alterations. A parallel myeloid NGS examination was conducted from BM aspirate in another department to exclude hematological malignancies. Both NGS examinations confirmed the IDH1 (c.395G > A; p.Arg132His) missense mutation. As an additional alteration, a BCL6 co-repressor (BCOR) (c.4087_4088delTG) frameshift, pathogenic mutation was detected from the BM aspirate (VAF: 52.91%). Furthermore, the IDH1 c.395G > A; p.Arg132His mutation was successfully detected from peripheral blood using NGS.

To detect possible further CNVs, MLPA analysis was performed from the primary tumor and BM aspirate. The MLPA result of the primary tumor was del(1p), dup(1q), del(4p)/del(4q), dup(8p), dup(11q), del(13q), del(18p/del(18q), del(19q), while for the metastasis it was the following: del(1p), dup(1q), dup(7p)/dup(7q), del(10p)/del(10q), del(18p)/del(18q), del(19q), dup(20q), dup(21q). These results confirm 1p/19q codeletion in both the primary tumor and the metastatic samples. Additionally, monosomy of chromosome 18 (del(18p)/del(18q)) was detected in both primary and metastatic tumors. Remarkably, chromosome 7 trisomy and chromosome 10 monosomy (+ 7/-10) were detected only in the metastasis. The latter molecular alterations are frequently seen in glioblastoma which may indicate a molecular progression of the case.

## Discussion

Extraneural metastases originating from primary CNS tumors are exceedingly rare. Among these, oligodendrogliomas present the lowest risk for extracerebral dissemination. The most common primary CNS tumors to metastasize outside the CNS include glioblastoma, medulloblastoma and ependymoma. This phenomenon of limited extracerebral metastasis can be attributed to several interrelated factors. The absence of a lymphatic system, the presence of the blood-brain barrier, and the lack of a sufficiently hostile CNS environment to select out metastatic clones have all been hypothesized as explanatory contributors. Additionally, the relatively shortened lifespan of most patients afflicted with these tumors may play a role in this rarity [5, 6, 7]. Furthermore, considering the immunologically privileged status of the CNS, glioma cells may harbor specific membrane antigens that could trigger cytotoxic responses from the patient’s lymphocytes during hematogenous dissemination. This robust immunosurveillance could significantly hinder the establishment of micrometastases or their progression into overt metastatic lesions [4].

Drawing from a systematic review encompassing 116 recorded cases of metastatic gliomas, 7 of which were metastatic oligodendrogliomas, the most prominent determinant of distant extracerebral dissemination is the direct access of the tumor to the extra meningeal tissues, often following craniotomy [4]. Many additional factors have since been associated with extracerebral metastatic recurrence, including repeated intracranial surgery, shunt surgery, or prolonged survival following chemoradiotherapy [7]. Notably however, in many cases, post-surgical access of the recurrent tumor growth to the extra meningeal tissues has been demonstrably independent of the site of operation. As observed in the systematic review, direct extrameningeal extension of the tumor over the cranial convexity was as prevalent in sites distant from the operative flap as through the flap itself. As such, among the 7 reviewed cases of metastatic oligodendrogliomas, the cranial convexity served as the site of exit for 3 cases [4]. Subsequent herniation of the cerebral cortex through the dura in the middle fossa was a frequent find complication due to the increasing intracranial pressure caused by both direct tumor expansion and cerebral edema. Therefore, it is clear that surgical interventions could, in many cases, increase the risk of hematogenous or lymphogenous metastasis by disrupting the blood-brain barrier or by granting tumor cells access to the dura mater or scalp. However, the development of extracranial metastases has been also documented in nonsurgical patients indicating that, in some instances, hematogenous spread of tumor cells can take place spontaneously [4, 6].

A focused literature review of 61 reported cases of extraneural metastases oligodendrogliomas has identified bone and bone marrow as the most frequent extraneural metastatic sites; 96% of which occurred after surgical excision of the primary tumor [5]. Clinically, however, metastatic bone marrow involvement results in an especially challenging paradigm as it causes cytopenia which then results in the cessation of the chemotherapy. Consequently, this allows the tumor to persistently grow, thereby exacerbating the bone marrow involvement and cytopenia.

While the aforementioned literature reviews have revealed critical insight into such a rare phenomenon, significant advancements have occurred in the methodology of diagnostic and prognostic assessment of metastatic oligodendrogliomas since that time. Prior to such technological advancements, the diagnosis and grading of oligodendrogliomas had been based solely on histopathology. Recently identified molecular markers, including the non-reciprocal translocation resulting in a 1p/19q codeletion, and mutations in the IDH1 or IDH2 gene, have significantly enhanced our understanding and characterization of oligodendrogliomas [8]. Currently, according to the WHO classification, diagnosis of oligodendrogliomas requires demonstration of IDH mutation by IDH1 (R132H) immunohistochemistry and/or sequencing of the IDH1/ IDH2 gene, as well as demonstration of 1p/19q codeletion by FISH, chromogenic in situ hybridization, or molecular genetic testing [1].

Although many cases of metastatic oligodendrogliomas have been reported, the majority do not have molecular confirmation for both the primary and metastatic lesions. A thorough search yielded only four published cases of metastatic oligodendroglioma with confirmed 1p/19q codeletion in both primary and metastatic lesions, verified by molecular studies.

In 2004, a recurrent oligodendroglioma, WHO grade 3, with metastasis to the right parotid gland was confirmed by capillary electrophoresis showing 1p/19q loss of heterozygosity (LOH) in both the intracranial tumor and the parotid gland lesion [8]. In 2010, an oligodendroglioma, WHO grade 3, with extensive extracranial metastases to the vertebrae, cervical lymph nodes, spinal dura mater, thymus, and chest wall was confirmed by FISH analysis showing 1p/19q codeletion in both the primary tumor and the cervical metastasis [9]. A 2019 case reported scalp metastases from a primary oligodendroglioma, WHO grade 3 with molecular confirmation of 1p/19q codeletion in both lesions [10]. The most recent case in 2020 involved multiple bone and bone marrow metastases from a 1p/19q-codeleted and IDH2-mutant oligodendroglioma, WHO grade 3, confirmed by MLPA studies [11]. These examples highlight the need for molecular studies when reporting such cases, especially given the potential for misdiagnosis of oligodendroglioma-like glioblastomas.

In this case report, NGS and FISH studies along with histopathology of both BM and CNS biopsy samples were used to define and classify the tumor and confirm metastasis. Additional cytological and molecular markers of indeterminate diagnostic and prognostic value, such as BCOR (c.4087_4088delTG) frameshift mutation and chromosomal instability, have been identified in our case study. Notably, the combination of gain of chromosome 7 and loss of chromosome 10 (+ 7/-10) is a characteristic molecular signature for IDH-wild-type glioblastoma and may indicate molecular progression of the oligodendroglioma. Furthermore, the presence of many copy number variations (CNVs) indicates chromosomal instability and is more commonly observed in high-grade tumor types with associated earlier tumor recurrence and a less favorable prognosis [12, 13, 14, 15]. Specifically, the presence of > 10 CNVs has been associated with poorer survival in CNS WHO grade 2 or 3 oligodendrogliomas [16]. On the other hand, BCOR has been acknowledged as a recurrently mutated gene in a subset of pediatric CNS tumors as well as in the recently defined CNS high-grade neuroepithelial tumor - BCOR altered, which is characterized by internal tandem duplication in exon 15 of the BCOR gene [17, 18]. However its potential relevance in the prognosis of oligodendrogliomas remains unexplored.

Furthermore, minimally invasive diagnostic practices such as liquid biopsies have become increasingly integrated into clinical practices. Harvesting circulating tumor DNA (ctDNA) derived from necrotic tumor cells from biofluids such as plasma, CSF, and urine has been an effective alternative for CNS tumor characterization and monitoring disease progression. The liquid biopsy analysis obtained in this case study further proves the effectiveness of such alternative diagnostic methods in metastatic oligodendrogliomas via the detection of IDH1 c.395G > A; p.Arg132His gene mutation.

This case study presents an example of a metastatic oligodendroglioma with bone marrow metastasis. Due to its rare occurrence, it can pose a diagnostic challenge, and the possibility of this should be considered in cases of known malignant central nervous system tumors. In metastatic oligodendroglioma, involvement of the bone marrow is a relatively common localization. In our case, beyond the diagnostic challenge, therapeutic management was complicated due to cytopenia, necessitating a modification in the treatment approach. The value of our case lies in the comprehensive molecular analysis conducted for both the primary tumor and the metastasis. We identified molecular progression in the metastasis that, to the best of our knowledge, has not been previously described in metastatic oligodendroglioma.

## Data Availability

No datasets were generated or analysed during the current study.

## Abbreviations

CNS: central nervous system
IDH: isocitrate dehydrogenase
TERT: telomerase reverse transcriptase
WHO: world health organisation
CT: computed tomography
MRI: magnetic resonance imaging
PCV: procarbazine hydrochloride, lomustine (CCNU), and vincristine sulfate
SRT: stereotactic radiation therapy
TMZ: temozolomide
BM: bone marrow
FFPE: formalin fixed paraffin embedded
GFAP: glial fibrillary acidic protein
NGS: next generation sequencing
SNV: single nucleotide variant
FISH: Fluorescent in situ hybridization
MLPA: Multiplex ligation-dependent probe amplification
CNV: copy number variation
BCOR: BCL6 interacting co-repressor
NCAM: neural cell adhesion molecule
ctDNA: circulating tumor deoxyribonulceic acid
CSF: cerebrospinal fluid
HE: hematoxylin and eosin
